# Readiness and Willingness to Provide Immunization Services after Pilot Vaccination Training: A Survey among Community Pharmacists Trained and Not Trained in Immunization during the COVID-19 Pandemic in Poland

**DOI:** 10.3390/ijerph18020599

**Published:** 2021-01-12

**Authors:** Piotr Merks, Urszula Religioni, Krzysztof Bilmin, Jedrzej Lewicki, Marta Jakubowska, Anna Waksmundzka-Walczuk, Aleksandra Czerw, Agnieszka Barańska, Joanna Bogusz, Katarzyna Plagens-Rotman, Dariusz Świetlik, Ewelina Drelich, Damian Świeczkowski, Jane Lambert, Miłosz Jaguszewski, Grzegorz Juszczyk, Bander Balkhi, Regis Vaillancourt

**Affiliations:** 1 Faculty of Medicine, Collegium Medicum, Cardinal Stefan Wyszyński University, 01-938 Warsaw, Poland; piotrmerks@googlemail.com (P.M.); kbilmin@wp.pl (K.B.); 2 Department of Pharmaceutical Technology, Faculty of Pharmacy, Collegium, Medicum in Bydgoszcz, 85-089 Bydgoszcz, Poland; ewelina.drelich@pgf.com.pl; 3 Collegium of Business Administration, Warsaw School of Economics, 02-513 Warsaw, Poland; 4 Polish Pharmaceutical Group, 91-342 Łódź, Poland; jedrzej.lewicki@pgf.com.pl (J.L.); marta.jakubowska@pgf.com.pl (M.J.); jamilosz@gmail.com (M.J.); 5 Solec Hospital, 00-382 Warsaw, Poland; aw.waksmundzka@gmail.com; 6 Department of Economics and Medical Law, Medical University of Warsaw, 00-791 Warsaw, Poland; ola_czerw@wp.pl; 7 Department of Economic and System Analysis, National Institute of Public Health-National Institute of Hygiene, 00-791 Warsaw, Poland; 8 Department of Computer Science and Medical Statistics with the Studio of Remote Learning, Medical University of Lublin, 20-954 Lublin, Poland; agnieszkabaranska@umlub.pl; 9 Department of Epidemiology, National Institute of Public Health-National Institute of Hygiene, 00-791 Warsaw, Poland; joanna.bogusz@go2.pl; 10 Hipolit Cegielski State University of Applied Sciences, 62-200 Gniezno, Poland; plagens.rotman@gmail.com; 11 Department of Biostatistics and Neural Networks, Medical University of Gdansk, Debinki 1, 80-211 Gdansk, Poland; dswietlik@gumed.edu.pl; 12 First Department of Cardiology, Medical University of Gdansk, Debinki 7, 80-211 Gdansk, Poland; d.swieczkowski@o2.pl; 13 Emergency Care Gateway (ECG), Milton Keynes MK13 9AP, UK; Jane@ecgtraining.co.uk; 14 Department of Public Health, Medical University of Warsaw, 02-097 Warsaw, Poland; grzegorz.juszczyk@wum.edu.pl; 15 National Institute of Public Health-National Institute of Hygiene, 00-791 Warsaw, Poland; 16 Medication Safety Research Chair, College of Pharmacy, King Saud University, Riyadh 4545, Saudi Arabia; bbalkhi@ksu.edu.sa; 17 College of Pharmacy-Department of Clinical Pharmacy, King Saud University, Riyadh 4545, Saudi Arabia; 18 Children’s Hospital of Eastern Ontario, Ottawa, ON K1H 8L1, Canada; rvaillancourt@cheo.on.ca

**Keywords:** community pharmacists, vaccines, immunization services, Poland, Pharmacist Without Borders project

## Abstract

Background: Immunization rates among the adult population in Poland are below desired targets, urging the need to expand this service in the community. During the COVID-19 pandemic, the ultimate goals for limiting the spread of the infection are vaccines against SARS-CoV-2. Pharmaceutical companies are in a race for the fastest possible way to deliver vaccines. Community pharmacists in Poland are recognised as an accessible yet underutilised group of medical professionals. Therefore, involving pharmacists in vaccinations may have beneficial results for the healthcare system. Objectives: The objectives of this study were to assess the readiness and willingness of community pharmacists following the Pharmacist Without Borders project who had either been trained or not in providing immunization services, and to identify the factors that may support the implementation of such services in Poland. Methods: This study was conducted among pharmacists between February and August 2020 in Poland. A survey was developed to determine their readiness to provide vaccination services in their pharmacies, to recognise any barriers to vaccinations, as well as the factors necessary to implement vaccination services in Polish pharmacies. Results: A total of 1777 pharmacists participated in the study, comprising 127 (7.1%) pharmacists trained in vaccinations during the Pharmacists Without Borders project and 1650 (92.9%) pharmacists not participating in the workshops. Pharmacists participating in the workshops more often indicated that providing vaccinations in community pharmacies would improve the overall vaccination rate (*p* = 0.0001), and that pharmacists could play an important role in advertising and promoting vaccinations (*p* = 0.0001). For the pharmacists not participating in the workshops, they indicated to a much greater extent possible barriers affecting the readiness to provide vaccinations in pharmacies. They most often pointed out that vaccination services would result in a significant workload increase (*p* = 0.0001), that pharmacies were not adapted to immunization, and that there were not enough training courses for pharmacists (*p* = 0.0001). Conclusion: The pharmacists working in community pharmacies indicated many advantages of vaccinations in pharmacies. This study identified barriers to the introduction of vaccinations and factors necessary to implement these services in pharmacies. The pharmacists trained during the immunization programme of the Pharmacists Without Borders project showed a greater readiness to provide immunization services.

## 1. Introduction

The development of vaccinations has contributed to a significant decrease in infectious diseases worldwide [1]. Not only are infectious diseases that causes of high mortality and incidence rates, but also have a major contribution of social and economic losses [2,3].

Vaccinations are particularly important when the consequences are considered not only individually but also locally and globally, where common use of vaccinations in just some groups of people can result in general herd immunity to infectious diseases, lowering the incidence and exacerbating eradication. However, overall herd immunity depends on a high vaccination coverage rate [1].

Despite the recommendations in favour of vaccinations, the burden of diseases that can be prevented by vaccinations remains high in many countries. This situation is especially important among senior citizens, leading to high mortality rates as well as the necessity to use resources and bear costs that could be avoided by vaccinations [4]. For example, it is predicted that the next 30 years will see an increase in the annual economic burden of four diseases in the United States among people aged >50 years that could be prevented by vaccinations (flu, whooping cough, herpes zoster, and pneumococcal disease) from 35 to 49 billion dollars, resulting in one million new deaths connected with these diseases [5]. Infections can have a negative impact on patient with chronic diseases and can worsen their symptoms, which can lead to hospitalisation and higher healthcare costs [5,6].

One of the most recommended vaccinations is for the flu. Costs of treating the flu increase with patient age and comorbidities [7]. Despite the possibility of getting vaccinated, Poles have had a low interest in flu vaccinations for many years, with the vaccination coverage rate one of the lowest in Europe [8]. One barrier can be insufficient access to vaccinations, indicated in patient studies in other countries [9,10], where pursuant to the law, vaccinations in Poland can only be administered by nurses or doctors after meeting specified criteria, and only doctors can qualify patients for vaccinations [11]. In many European countries, vaccinations are administered by nursing staff and pharmacists. Pharmacists are perceived as a highly qualified and accessible professional group [10]. Specially trained people vaccinating patients in clinics and community pharmacies are increasingly common. As the experiences of other countries show, the implementation of vaccinations in pharmacies increases the vaccination coverage rate, significantly reducing the workload of the healthcare system [12].

Following European trends, educational workshops were organised for pharmacists in Poland—“Pharmacists Without Borders” [13]. Pharmacists participated in a course that prepared them to administer vaccinations in pharmacies—“Immunoprevention of Selected Infectious Diseases”. The workshops were organised in cooperation with Emergency Care Gateway ECG—an accredited training provider for the British healthcare (NHS).

The objective of the workshop was to update and deepen knowledge in vaccinology, with particular emphasis on the types of vaccination, their safety, routes of administration, and storage. The issues covered in the workshops included safe and correct administration of intramuscular injections, as well as diagnosing and treating patients with symptoms of anaphylaxis and cardiac arrest.

After completing the course and particular modules, the participants of the “Pharmacists Without Borders” workshop received European certification certifying their knowledge in the field of vaccination and injections in pharmacies (*ISO 9001 Quality Management), accredited by the Royal Pharmaceutical Society of Great Britain and Collegium Medicum at the Ludwik Rydygier Medical College in Bydgoszcz.

The proposal of including vaccination in the tasks of pharmaceutical care had been indicated in the Polish State Medicine Policy for the period 2018–2022 [14], yet there has been no assessment of the readiness of pharmacists to provide this type of service in pharmacies. Therefore, the main objective of this paper is to assess the willingness and readiness of pharmacists to administer vaccinations in community pharmacies in Poland among two groups of pharmacists (those trained and not in providing vaccination services in the Pharmacists Without Borders project). In addition, the study aims to identify any barriers to the implementation of vaccination in pharmacies, as well as factors necessary for the implementation of this type of pharmaceutical service in Poland.

## 2. Methods

The study was anonymous and was conducted among pharmacists working in community pharmacies in Poland between February and August 2020. The readiness and willingness of pharmacists to provide vaccination services were assessed by means of a questionnaire specifically designed for this study on the basis of a study conducted in the Saudi Arabia [15], and in order to adjust it to the Polish healthcare system, it was reviewed by academic experts. The final version of the survey was translated to Polish. The study was approved by Ethical Board at Collegium Medicum in Bydgoszcz, Poland. The questionnaire was distributed among closed pharmacist groups through professional forums that belonged to the Pharmacy Chamber and Trade Union of Pharmacy Workers via personal mailing for each organization’s members.

The data collected included data on the pharmacists (age, professional title, and job seniority) and the pharmacies (type of pharmacy), as well as data relevant for the purposes of the survey (justification of readiness to provide vaccination services in pharmacies, barriers to vaccination, and factors necessary to implement vaccination services in pharmacies). Each item of the questionnaire was assessed using a 5-point Likert compliance scale (1—definitely no; 5—definitely yes).

All statistical calculations were performed using the data analysis software STATISTICA version 12.0 (StatSoft Inc. 2014, Tulsa, OK, USA) and MS Excel. Qualitative variables were presented by the number and percentage values and Chi-square independence tests (respectively, using Yates correction for cell sizes below 10, checking Cochran conditions, exact Fisher test). To assess the impact of basic characteristics on vaccination readiness, barriers, and pharmacists’ vaccination needs in pharmacies, the univariate and multivariate regression (multiple linear regression) analysis were used. In all calculations the level of statistical significance was taken at *p* = 0.05.

## 3. Results

The study involved 1777 pharmacists, comprising 127 (7.1%) pharmacists who had participated in the Pharmacists Without Borders workshops, and 1650 (92.9%) pharmacists who had not participated. Most respondents held an MPharm degree, were mainly aged 31–40 (39.6%), and most often with less than 10 years’ job seniority (40.7%). The pharmacists worked in both independent pharmacies (51.7%) and pharmacy chains (48.3%). Despite no significant differences in age, job seniority and type of pharmacy of the pharmacists participating and not participating in the Pharmacists Without Borders workshops, a higher percentage of pharmacists with a PhD(Pharm) attended the workshops than did not (Table 1).

As far as community pharmacies are concerned, the self-assessments of the pharmacists’ knowledge of vaccinations and their indications varied, but the responses of the groups of pharmacists after and without training did not differ (*p* = 0.8704). Among the statements on the justification of their readiness to provide vaccination services in community pharmacies, the following were most frequently indicated in the entire study group: pharmacists from community pharmacies are easily accessible to patients, and pharmacists from community pharmacies can play an important role in advertising and promoting vaccinations. The pharmacists participating in the Pharmacists Without Borders workshops indicated more often than not that vaccinations administered in community pharmacies would improve the overall vaccination coverage rate (*p* = 0.0001), including the vaccination coverage rate in particular groups, e.g., seniors (*p* = 0.0001). The pharmacists participating in the workshops more often indicated that a vaccination project could be cost effective (*p* = 0.0001) and that pharmacists could play an important role in advertising and promoting vaccinations (*p* = 0.0001) (Table 2).

Justification of readiness to provide vaccination services in pharmacies in relation to participants’ basic characteristics was analysed using univariate and multivariate regression analysis. Both models were statistically significant. In the univariate regression model, significantly greater readiness for vaccination was demonstrated for pharmacists up to 30 years of age (*p* = 0.0021), with less than ten years of work experience (*p* = 0.0272) and completed Pharmacists Without Borders workshop (*p* = 0.0001). In the multivariate regression model, significantly greater readiness for vaccination was demonstrated for pharmacists up to 30 years of age (*p* = 0.0273) and completed the Pharmacists Without Borders workshop (*p* = 0.0001) (Table 5).

Among the barriers affecting the readiness to provide vaccination services in pharmacies, the pharmacists pointed out, above all, the following statements: providing vaccinations will add more work to pharmacists, there are not enough training courses for pharmacists, pharmacies are not adjusted to provide these services. The pharmacists not participating in the Pharmacists without Borders workshops indicated to a much greater extent all possible barriers affecting the readiness to provide vaccinations in pharmacies (in each statement *p* = 0.0001) (Table 3).

Barriers affecting the pharmacists’ readiness to provide vaccination services in relation to basic characteristics of participants were analyzed using univariate and multivariate regression analysis. Both models were statistically significant.

In the univariate regression model, significantly lower barriers to vaccination were demonstrated for pharmacists up to 30 years of age (*p* = 0.0376), with the title of Ph.D. in pharmacy (*p* = 0.0242), working in independent pharmacies (*p* = 0.0165), and completed workshops Pharmacists without Borders (*p* = 0.0001). On the other hand, significantly greater barriers to vaccination were demonstrated for pharmacists with a master’s degree in pharmacy (*p* = 0.0285), working in chain pharmacies (*p* = 0.0165), and with the lack of completion of the Pharmacists without Borders workshop (*p* = 0.0001).

In the multivariate regression model, significantly lower vaccination barriers were shown for pharmacists up to 30 years of age (*p* = 0.0158), working in independent pharmacies (*p* = 0.0095), and with completed Pharmacists without Borders workshops (*p* = 0.0001). On the other hand, significantly greater barriers to vaccination were shown for pharmacists who did not complete the Pharmacists Without Borders workshop (*p* = 0.0001) (Table 5).

Among the factors necessary for the implementation of vaccination services in community pharmacies, pharmacists from the entire study group indicated above all that the possibility to designate rooms for administering vaccinations in pharmacies, relevant remuneration or reimbursement for vaccination services, and cooperation between pharmacists and healthcare centres were necessary (Table 4).

The pharmacists participating in the Pharmacists Without Borders workshops indicated much more often that there was a need for more university education and training courses for pharmacists in the administration of vaccinations (*p* = 0.0463), and that patients expect the implementation of vaccinations administered by pharmacists (*p* = 0.0001).

On the other hand, those pharmacists who had not participated in the workshops more often stressed that in order to implement vaccination services, it was necessary to be able to designate special rooms for administering vaccinations in pharmacies (*p* = 0.0002) and to employ more pharmacists in pharmacies (*p* = 0.0002).

Essential factors to implement vaccination services in pharmacies in relation to basic characteristics of participants were analyzed using univariate and multivariate regression analysis. Both models were statistically significant. In the univariate regression model, significantly greater needs were demonstrated by pharmacists up to 30 years of age (*p* = 0.0001), with the title of MSc in pharmacy (*p* = 0.0250), working in chain pharmacies (*p* = 0.0001), and less than ten years of work experience (*p* = 0.0001). On the other hand, significantly lower needs were indicated by pharmacists aged 41–50 (*p* = 0.0020) and over 50 (*p* = 0.0001), working in independent pharmacies (*p* = 0.0001) and with over ten years of work experience. The multivariate regression model showed significantly lower needs indicated by pharmacists aged over 50 (*p* = 0.0459) and working in independent pharmacies (*p* = 0.0049) (Table 5).

## 4. Discussion

To the best of our knowledge, this study is the first aimed at assessing the readiness of pharmacists to administer vaccinations in community pharmacies in Poland. Our research shows that pharmacists up to 30 years of age, with less than 10 years of experience in the profession and participating in Pharmacists Without Boarders workshops indicate the greatest readiness to perform vaccinations. According to the pharmacists, the most important elements influencing the readiness to administer vaccinations in pharmacies included the ease of access for patients and the role that they can play in advertising and promoting vaccinations. The pharmacists participating in the Pharmacists Without Borders workshops were much more likely to point out that providing vaccinations in community pharmacies would improve the overall vaccination coverage rate. This may be due to the higher level of knowledge and skills acquired during the workshops. Similar results were shown by other studies, e.g., those conducted in the United States [16] and Saudi Arabia [15]. In both cases, the pharmacists emphasised easy access to vaccinations and the impact on the increased vaccination coverage rate. Long opening hours, a small number of waiting persons, and a large number of pharmacies make the pharmacy a comfortable place to receive a vaccination. In addition, the extensive knowledge of pharmacists, who are a professional group not fully effectively used in healthcare, makes the implementation of vaccinations in pharmacies a good solution for the healthcare system [10,17,18].

Many studies indicate the positive effects of implementing vaccinations in community pharmacies. The results of these studies prove that pharmacists increase the availability of vaccinations, accelerate immunization, effectively educate patients, affect the vaccination coverage rate, prevent new cases of diseases, and by reducing the number of diseases or complications, they bring savings to the healthcare system [19,20,21,22].

Experiences in such countries as England, Portugal, and the United States prove the benefits of vaccinations for both patients and the healthcare system [23]. Similarly, in Canada, following the implementation of vaccinations administered by pharmacists, the proportion of people vaccinated in the general population has increased significantly [24].

International organisations such as the American Public Health Association (APHA) and the International Pharmaceutical Federation (FIP) encourage pharmacists to get involved in administering vaccinations [25,26]. The World Health Organisation (WHO) has adopted the Global Vaccine Action Plan (GVAP) [27], whose main objective is to increase access to vaccinations for people around the world to reduce the incidence and mortality of infectious diseases. The role of pharmacists is highlighted in particular with regard to flu vaccination. The Global Influenza Strategy 2019–2030, developed by the World Health Organisation (WHO), emphasises the significant role of vaccinations in minimising the effects of seasonal flu [28].

Many countries have permitted pharmacists to administer vaccinations. Vaccinations administered by pharmacists are common in community pharmacies in 13 European countries, including the United Kingdom, Norway, Greece, Portugal, Estonia, and other countries want to join in these activities [12].

This study identified potential barriers to the implementation of vaccinations in Polish pharmacies. The pharmacists mainly pointed out the increased workload, insufficient training for pharmacists, and the fact that pharmacies were not adjusted to provide vaccinations. Much fewer barriers were indicated by pharmacists under 30, with the Ph.D. title, working in independent pharmacies. What is more, the pharmacists not participating in the Pharmacists Without Borders workshops pointed out various barriers to a much greater extent. The lack of relevant training courses as a barrier to providing vaccinations in pharmacies was also pointed out by researchers in Saudi Arabia [15].

Other studies on patients’ attitudes and beliefs about vaccinations in community pharmacies indicate that patients express the need to use such services and accept that pharmacists may administer vaccinations. Patient concerns about these services relate to uncertainty about the skills of the pharmacist or the lack of a place for administering vaccinations in pharmacies where patient privacy can be maintained [29].

A Canadian study suggests that pharmacists’ main concerns about vaccinations in pharmacies are logistical issues, while public concerns relate to uncertainties about the safety of vaccinations administered in pharmacies, keeping of medical records by pharmacists, and the cost of such vaccinations. Patients in Canada expressed the opinion that the cost of vaccination administered in pharmacies should not be higher than the cost of vaccinations administered in another facility [30].

When implementing vaccinations in pharmacies, patient-related barriers influencing the overall low vaccination coverage rate should also be considered. Literature highlights low patient knowledge, uncertainty about vaccination safety, the need to travel to facilities at inconvenient times to get vaccinated, or the need to wait a long time for vaccination [10,31,32].

Taking into account the above, it seems that the implementation of vaccinations in pharmacies will minimise many barriers for patients. Convenient locations and open hours, the possibility of educating patients and explaining any disturbing information, may effectively increase the patient vaccination coverage rate.

This situation is particularly important given the fact that flu vaccination rates in those over 65 years of age in Poland are under 10%. By comparison, this level is 72% in the United Kingdom and 68.7% in the United States [8]. Additionally, flu vaccinations in Poland are performed almost exclusively in the oldest age groups (75% of vaccinations are administered to patients over 65 years of age) [33].

Among the factors necessary for the implementation of vaccination services in community pharmacies, pharmacists from the entire study group indicated above all that the possibility to designate rooms for administering vaccinations in pharmacies, relevant remuneration or reimbursement for vaccination services, and cooperation between pharmacists and healthcare centres were necessary. Pharmacists aged up to 30, with the MSc title in pharmacy, working in chain pharmacies, and with less than 10 years’ work experience, indicated much greater needs.

Barriers on the part of the healthcare system, e.g., no relevant vaccination regulations, no extensive opportunities for education related to vaccinations, and a lack of access to patient medical data by pharmacists, should also be taken into account [18,32]. In Poland, at the end of 2018, there were 12.9 thousand pharmacies [34], with 1.85 pharmacists per pharmacy. This number is much smaller in comparison with other countries (the average for EU countries is 2.40 pharmacists per pharmacy) [35]. What is more, pursuant to the law, only a doctor can qualify a person for a vaccination, but vaccinations can be administered by a doctor, nurse or midwife, with appropriate qualifications.

Apart from thorough legal and organisational changes, a broad education of patients on vaccinations is necessary in Poland. In many countries, patients are satisfied with the availability of vaccinations in pharmacies. In Poland, patients’ opinions on providing vaccinations by pharmacists should be investigated.

If vaccinations are implemented in pharmacies, campaigns will be needed to inform patients of this possibility. The implementation of vaccinations as a pharmaceutical service will also require small-scale pilot programmes to assess the results and identify areas that can be improved when preparing larger-scale activities. Results of this study should be used while planning the immunization legislation in Poland.

In Poland, in mid-June 2020, a Coalition for Vaccination in Pharmacies was established, associating 3 organisations of doctors and pharmacists—the National Influenza Control Programme, the Supreme Pharmaceutical Chamber and the Trade Union of Pharmacy Workers. This idea is supported by the Supreme Pharmaceutical Chamber, which recognises that pharmacists can greatly increase patient knowledge of vaccinations and increase the level of vaccination against infectious diseases, especially the flu [36]. The implementation of vaccinations administered by suitably prepared and trained pharmacists will only be possible after setting detailed standards for such services and precise guidelines for vaccinations administered in pharmacies, including monitoring, reporting and responding to adverse events. It will also be necessary to establish strict educational requirements to be met by pharmacists to administer vaccinations.

In the prevention of infectious diseases, especially in the case of the flu, the Polish healthcare system requires the relief from burden that can be achieved by using pharmacists in the provision of preventive services. At the same time, these activities require relevant preparation of pharmacies in terms of sanitation and logistics. Pharmacists wishing to administer vaccinations must obtain appropriate qualifications, which many of them do not currently have. For this reason, as part of the Pharmacists Without Borders initiative, workshops have been organised to prepare pharmacists for administering vaccinations in pharmacies. Studies on the effects of vaccination education for pharmacists in Italy showed that pharmacists with greater knowledge of vaccinations were more willing to engage in this type of services [37]. Similarly, our own study has unequivocally confirmed that pharmacists participating in immunization workshops saw more benefits from vaccinations administered in pharmacies, and also indicated the lower strength of barriers to the implementation of vaccinations in the Polish healthcare system.

Limitations of the study

- The various data sources enabled to reach nearly 1800 responses from pharmacists among 28,000 registered pharmacists in Poland, strengthening the exploration of the research topic. The response rates in normal conditions are low.
- Voluntary self-administered questionnaire distributed to pharmacists who will be willing to provide pharmacist immunization services and interviews with flu vaccination trained pharmacists with the Pharmacist Without Boarders project could have caused participant bias as the response of trained pharmacist was lower than pharmacist who were not trained.
- This study was conducted in the whole regions of Poland, but the most active region in response was the Mazovian region, where pharmacy chamber is most innovative and open for new projects. Findings might differ in the other areas and local pharmacy jurisdictions due to differences in vaccination service opinions in pharmacies.

## 5. Conclusions

Protective vaccinations are one of the most effective methods of combating infectious diseases. The widespread use of vaccinations makes it possible to significantly reduce the spread of many diseases that contribute to serious health consequences, and often death. In this context, the significant reduction in social and economic costs of diseases from vaccinations should also be highlighted. However, the reduction in vaccination coverage rates observed in recent years may lead to the re-emergence of many diseases even if the disease in question has already been eradicated in a given area. Thus, ensuring the provision of vaccinations for the most important population groups, from the point of view of the entire community, should be one of the most important activities of institutions dealing with public health.

Community pharmacies in Poland are facilities where vaccinations can be administered. This study shows that pharmacists are, to a large extent, prepared to administer vaccinations, although they indicate the need to increase their immunization knowledge. Educational programmes such as Pharmacists Without Borders, can to a significant extent prepare pharmacists to administer vaccinations in community pharmacies. In addition to providing pharmacists with the relevant education, it is necessary to prepare standards as well as a legal framework for the provision of such services. These actions are particularly important during the COVID-19 pandemic as pharmacists can have a significant impact on the vaccination coverage rate of patients, and thus take an active part in combating infectious diseases.

## Figures and Tables

**Table 1:** Basic characteristics of the pharmacists participating in the Pharmacists Without Borders workshops (Group Yes) and the pharmacists not participating in the Pharmacists Without Borders workshops (Group No) by age, title, type of pharmacy, and job seniority.

Variable	Workshop Participants (*n* = 127)	Pharmacist Not Participating in the Workshop (*n* = 1650)	Total (*n* = 1777)	*p*-Value
Age				0.9389 ^1^
less than 30 years	27 (21.3%)	366 (22.2%)	393 (22.1%)	
31–40 years	48 (37.8%)	655 (39.7%)	703 (39.6%)	
41–50 years	30 (23.6%)	359 (21.8%)	389 (21.9%)	
50 years and over	22 (17.3%)	270 (16.4%)	292 (16.4%)	
Title				0.0001 ^1^
MPharm Intern	1 (0.8%)	22 (1.3%)	23 (1.3%)	
MPharm	115 (90.6%)	1602 (97.1%)	1717 (96.6%)	
PhD(Pharm)	11 (8.7%)	26 (1.6%)	37 (2.1%)	
Type of pharmacy				0.5406 ^1^
independent	69 (54.3%)	850 (51.5%)	919 (51.7%)	
chain	58 (45.7%)	800 (48.5%)	858 (48.3%)	
Job seniority				0.8503 ^1^
less than 10 years	50 (39.4%)	673 (40.8%)	723 (40.7%)	
10–20 years	43 (33.9%)	553 (33.5%)	596 (33.5%)	
21–30 years	25 (19.7%)	283 (17.2%)	308 (17.3%)	
more than 30 years	9 (7.1%)	141 (8.5%)	150 (8.4%)	

^1^ Chi^2^ test.

**Table 2:** Justification of readiness to provide vaccination services in pharmacies by the pharmacists participating and not participating in the Pharmacists Without Borders workshops.

Possible Answers	After Training (*n* = 127)	Without Training (*n* = 1650)	Total (*n* = 1777)	*p*-Value
	Pharmacists from community pharmacies have good knowledge of vaccinations and their indications			
definitely no	11 (8.7%)	174 (10.5%)	185 (10.4%)	
rather no	40 (31.5%)	543 (32.9%)	583 (32.8%)	
I don’t know	11 (8.7%)	144 (8.7%)	155 (8.7%)	0.8704 ^1^
rather yes	51 (40.2%)	646 (39.2%)	697 (39.2%)	
definitely yes	14 (11.0%)	143 (8.7%)	157 (8.8%)	
	Pharmacists from community pharmacies are easily accessible to patients			
definitely no	6 (4.7%)	38 (2.3%)	44 (2.5%)	0.0001 ^1^
rather no	6 (4.7%)	126 (7.6%)	132 (7.4%)	
I don’t know	3 (2.4%)	31 (1.9%)	34 (1.9%)	
rather yes	27 (21.3%)	710 (43.0%)	737 (41.5%)	
definitely yes	85 (66.9%)	745 (45.2%)	830 (46.7%)	
	Vaccinations administered in community pharmacies will improve the overall vaccination coverage rate			
definitely no	13 (10.2%)	241 (14.6%)	254 (14.3%)	0.0001 ^1^
rather no	12 (9.4%)	445 (27.0%)	457 (25.7%)	
I don’t know	11 (8.7%)	352 (21.3%)	363 (20.4%)	
rather yes	34 (26.8%)	359 (21.8%)	393 (22.1%)	
definitely yes	57 (44.9%)	253 (15.3%)	310 (17.4%)	
	Vaccinations administered in community pharmacies will improve the vaccination coverage rate in particular groups of patients, e.g., seniors			
definitely no	14 (11.0%)	253 (15.3%)	267 (15.0%)	0.0001 ^1^
rather no	12 (9.4%)	431 (26.1%)	443 (24.9%)	
I don’t know	11 (8.7%)	332 (20.1%)	343 (19.3%)	
rather yes	39 (30.7%)	390 (23.6%)	429 (24.1%)	
definitely yes	51 (40.2%)	244 (14.8%)	295 (16.6%)	
	The project of vaccinations administered in community pharmacies is cost effective			
definitely no	19 (15.0%)	351 (21.3%)	370 (20.8%)	0.0001 ^1^
rather no	10 (7.9%)	272 (16.5%)	282 (15.9%)	
I don’t know	45 (35.4%)	765 (46.4%)	810 (45.6%)	
rather yes	28 (22.0%)	158 (9.6%)	186 (10.5%)	
definitely yes	25 (19.7%)	104 (6.3%)	129 (7.3%)	
	Pharmacists from community pharmacies can play a significant role in advertising and promoting vaccinations			
definitely no	10 (7.9%)	199 (12.1%)	209 (11.8%)	0.0001 ^1^
rather no	10 (7.9%)	282 (17.1%)	292 (16.4%)	
I don’t know	6 (4.7%)	157 (9.5%)	163 (9.2%)	
rather yes	31 (24.4%)	641 (38.8%)	672 (37.8%)	
definitely yes	70 (55.1%)	371 (22.5%)	441 (24.8%)	

^1^ Chi^2^ test.

**Table 3:** Barriers affecting the readiness to provide vaccination services by the pharmacists participating and not participating in the Pharmacists Without Borders workshops.

	After Training (*n* = 127)	Without Training (*n* = 1650)	Total (*n* = 1777)	*p*-Value
	Pharmacists have too much work and do not have time for vaccinations			
definitely no	9 (7.1%)	41 (2.5%)	50 (2.8%)	0.0001 ^1^
rather no	37 (29.1%)	205 (12.4%)	242 (13.6%)	
I don’t know	11 (8.7%)	74 (4.5%)	85 (4.8%)	
rather yes	37 (29.1%)	382 (23.2%)	419 (23.6%)	
definitely yes	33 (26.0%)	948 (57.5%)	981 (55.2%)	
	Providing vaccinations will add more work to pharmacists			
definitely no	1 (0.8%)	9 (0.5%)	10 (0.6%)	0.0001 ^1^
rather no	11 (8.7%)	55 (3.3%)	66 (3.7%)	
I don’t know	5 (3.9%)	17 (1.0%)	22 (1.2%)	
rather yes	59 (46.5%)	420 (25.5%)	479 (27.0%)	
definitely yes	51 (40.2%)	1149 (69.6%)	1200 (67.5%)	
	Patient safety when administering vaccinations is a problem			
definitely no	8 (6.3%)	38 (2.3%)	46 (2.6%)	0.0001 ^1^
rather no	51 (40.2%)	207 (12.5%)	258 (14.5%)	
I don’t know	7 (5.5%)	75 (4.5%)	82 (4.6%)	
rather yes	24 (18.9%)	330 (20.0%)	354 (19.9%)	
definitely yes	37 (29.1%)	1000 (60.6%)	1037 (58.4%)	
	There are not enough training courses for pharmacists			
definitely no	6 (4.7%)	21 (1.3%)	27 (1.5%)	0.0006 ^1^
rather no	6 (4.7%)	66 (4.0%)	72 (4.1%)	
I don’t know	7 (5.5%)	96 (5.8%)	103 (5.8%)	
rather yes	41 (32.3%)	353 (21.4%)	394 (22.2%)	
definitely yes	67 (52.8%)	1114 (67.5%)	1181 (66.5%)	
	Patients have less trust in pharmacists who provide these services			
definitely no	10 (7.9%)	34 (2.1%)	44 (2.5%)	0.0001 ^1^
rather no	18 (14.2%)	169 (10.2%)	187 (10.5%)	
I don’t know	36 (28.3%)	403 (24.4%)	439 (24.7%)	
rather yes	39 (30.7%)	542 (32.8%)	581 (32.7%)	
definitely yes	24 (18.9%)	502 (30.4%)	526 (29.6%)	
	Pharmacies are not adjusted to provide these services			
definitely no	8 (6.3%)	72 (4.4%)	80 (4.5%)	0.0001 ^1^
rather no	18 (14.2%)	86 (5.2%)	104 (5.9%)	
I don’t know	15 (11.8%)	38 (2.3%)	53 (3.0%)	
rather yes	33 (26.0%)	257 (15.6%)	290 (16.3%)	
definitely yes	53 (41.7%)	1197 (72.5%)	1250 (70.3%)	
	Conflicts with other specialists qualified to administer vaccinations are likely to occur			
definitely no	8 (6.3%)	15 (0.9%)	23 (1.3%)	0.0001 ^1^
rather no	22 (17.3%)	156 (9.5%)	178 (10.0%)	
I don’t know	15 (11.8%)	223 (13.5%)	238 (13.4%)	
rather yes	42 (33.1%)	534 (32.4%)	576 (32.4%)	
definitely yes	40 (31.5%)	722 (43.8%)	762 (42.9%)	
	There are concerns related to handling vaccinations, their storage and disposing of sharp objects			
definitely no	32 (25.2%)	200 (12.1%)	232 (13.1%)	0.0001 ^1^
rather no	46 (36.2%)	419 (25.4%)	465 (26.2%)	
I don’t know	6 (4.7%)	75 (4.5%)	81 (4.6%)	
rather yes	20 (15.7%)	416 (25.2%)	436 (24.5%)	
definitely yes	23 (18.1%)	540 (32.7%)	563 (31.7%)	
	Pharmacists do not feel comfortable when using needles			
definitely no	18 (14.2%)	113 (6.8%)	131 (7.4%)	0.0001 ^1^
rather no	36 (28.3%)	244 (14.8%)	280 (15.8%)	
I don’t know	29 (22.8%)	337 (20.4%)	366 (20.6%)	
rather yes	24 (18.9%)	390 (23.6%)	414 (23.3%)	
definitely yes	20 (15.7%)	566 (34.3%)	586 (33.0%)	

^1^ Chi^2^ test.

**Table 4:** Essential factors to implement vaccination services in pharmacies, indicated by the pharmacists participating and not participating in the Pharmacists Without Borders workshops.

Possible Answers	After Training (*n* = 127)	Without Training (*n* = 1650)	Total (*n* = 1777)	*p*-Value
	More university education and training courses for pharmacists in administering vaccinations are necessary			
definitely no	6 (4.7%)	57 (3.5%)	63 (3.5%)	0.0463 ^1^
rather no	2 (1.6%)	96 (5.8%)	98 (5.5%)	
I don’t know	3 (2.4%)	93 (5.6%)	96 (5.4%)	
rather yes	40 (31.5%)	392 (23.8%)	432 (24.3%)	
definitely yes	76 (59.8%)	1012 (61.3%)	1088 (61.2%)	
	Continuous training sessions and workshops in vaccinations are necessary			
definitely no	5 (3.9%)	61 (3.7%)	66 (3.7%)	0.4430 ^1^
rather no	3 (2.4%)	88 (5.3%)	91 (5.1%)	
I don’t know	3 (2.4%)	75 (4.5%)	78 (4.4%)	
rather yes	39 (30.7%)	497 (30.1%)	536 (30.2%)	
definitely yes	77 (60.6%)	929 (56.3%)	1006 (56.6%)	
	Relevant remuneration or reimbursement for vaccination services are necessary			
definitely no	4 (3.1%)	15 (0.9%)	19 (1.1%)	0.1734 ^1^
rather no	1 (0.8%)	17 (1.0%)	18 (1.0%)	
I don’t know	4 (3.1%)	54 (3.3%)	58 (3.3%)	
rather yes	15 (11.8%)	160 (9.7%)	175 (9.8%)	
definitely yes	103 (81.1%)	1404 (85.1%)	1507 (84.8%)	
	Patients expect the implementation of vaccinations administered by pharmacists			
definitely no	11 (8.7%)	250 (15.2%)	261 (14.7%)	0.0001 ^1^
rather no	26 (20.5%)	573 (34.7%)	599 (33.7%)	
I don’t know	37 (29.1%)	572 (34.7%)	609 (34.3%)	
rather yes	35 (27.6%)	192 (11.6%)	227 (12.8%)	
definitely yes	18 (14.2%)	63 (3.8%)	81 (4.6%)	
	The possibility to designate rooms for administering vaccinations in pharmacies is necessary			
definitely no	4 (3.1%)	13 (0.8%)	17 (1.0%)	0.0002 ^1^
rather no	5 (3.9%)	33 (2.0%)	38 (2.1%)	
I don’t know	1 (0.8%)	34 (2.1%)	35 (2.0%)	
rather yes	33 (26.0%)	249 (15.1%)	282 (15.9%)	
definitely yes	84 (66.1%)	1321 (80.1%)	1405 (79.1%)	
	It is necessary to employ more pharmacists in pharmacies to provide appropriate vaccination services			
definitely no	8 (6.3%)	19 (1.2%)	27 (1.5%)	0.0001 ^1^
rather no	18 (14.2%)	142 (8.6%)	160 (9.0%)	
I don’t know	16 (12.6%)	96 (5.8%)	112 (6.3%)	
rather yes	30 (23.6%)	410 (24.8%)	440 (24.8%)	
definitely yes	55 (43.3%)	983 (59.6%)	1038 (58.4%)	
	It is necessary to reduce the workload of technical tasks for pharmacists (e.g., entering invoices, verifying supplies) to save time for providing vaccination services			
definitely no	2 (1.6%)	40 (2.4%)	42 (2.4%)	0.4036 ^1^
rather no	14 (11.0%)	112 (6.8%)	126 (7.1%)	
I don’t know	7 (5.5%)	112 (6.8%)	119 (6.7%)	
rather yes	38 (29.9%)	468 (28.4%)	506 (28.5%)	
definitely yes	66 (52.0%)	918 (55.6%)	984 (55.4%)	
	Cooperation between pharmacists and health centres is necessary			
definitely no	2 (1.6%)	18 (1.1%)	20 (1.1%)	0.5489 ^1^
rather no	6 (4.7%)	60 (3.6%)	66 (3.7%)	
I don’t know	4 (3.1%)	100 (6.1%)	104 (5.9%)	
rather yes	34 (26.8%)	491 (29.8%)	525 (29.5%)	
definitely yes	81 (63.8%)	981 (59.5%)	1062 (59.8%)	
	The support of medical and nursing associations is necessary			
definitely no	4 (3.1%)	44 (2.7%)	48 (2.7%)	0.6641 ^1^
rather no	12 (9.4%)	112 (6.8%)	124 (7.0%)	
I don’t know	16 (12.6%)	271 (16.4%)	287 (16.2%)	
rather yes	37 (29.1%)	468 (28.4%)	505 (28.4%)	
definitely yes	58 (45.7%)	755 (45.8%)	813 (45.8%)	
	The possibility for pharmacists to specialise in providing vaccination services is necessary, e.g., obtaining a certificate confirming the ability to administer vaccinations			
definitely no	3 (2.4%)	76 (4.6%)	79 (4.4%)	0.0951 ^1^
rather no	4 (3.1%)	71 (4.3%)	75 (4.2%)	
I don’t know	2 (1.6%)	90 (5.5%)	92 (5.2%)	
rather yes	25 (19.7%)	382 (23.2%)	407 (22.9%)	
definitely yes	93 (73.2%)	1031 (62.5%)	1124 (63.3%)	

^1^ Chi^2^ test.

**Table 5:** Justification of readiness to provide vaccination services, barriers and essential factors to implement vaccination services in pharmacies in relation to basic characteristics of participants—univariate and multivariate regression analysis.

Factor	Justification of Readiness to Provide Vaccination Services in Pharmacies		Barriers Affecting the Readiness to Provide Vaccination Services by the Pharmacists		Essential Factors to Implement Vaccination Services in Pharmacies	
	Univariate	Multivariate	Univariate	Multivariate	Univariate	Multivariate
	*p*-Value	*p*-Value	*p*-Value	*p*-Value	*p*-Value	*p*-Value
Age						
less than 30 years	0.0021	0.0273	0.0376	0.0158	0.0001	0.1456
31–40 years	0.3321		0.1220		0.2541	
41–50 years	0.3766		0.9343		0.0020	0.1551
50 years and over	0.2387		0.8456		0.0001	0.0459
Title						
MPharm Intern	0.4324		0.2232		0.0250	0.1721
MPharm	0.1330		0.0285	0.3011	0.8926	
PhD(Pharm)	0.2009		0.0242	0.9354	0.0519	
Type of pharmacy						
independent	0.8766		0.0165	0.0095	0.0001	0.0049
chain	0.8766		0.0165	0.0095	0.0001	0.0049
Job seniority						
less than 10 years	0.0272	0.7201	0.5133		0.0001	0.9134
10–20 years	0.1763		0.4793		0.0052	0.2516
21–30 years	0.4122		0.7041		0.0023	0.5408
more than 30 years	0.6261		0.5733		0.0023	0.5408
Participation in the Pharmacists Without Borders workshop						
Yes	0.0001	0.0001	0.0001	0.0001	0.7609	
No	0.0001	0.0001	0.0001	0.0001	0.7609	

## Data Availability

The exact data can be obtained from the corresponding author.
